# Implementing a “resect and discard” strategy using a characterization mobile app: toward a more sustainable endoscopy practice

**DOI:** 10.1055/a-2616-8361

**Published:** 2025-06-26

**Authors:** Noémie Costaouec, Pierre Lafeuille, Orlando Chuquimia, Mathilde Chatain, Victoria Nurcelli, Elena De Cristofaro, Mathieu Pioche

**Affiliations:** 1 Department of Endoscopy and Hepatogastroenterology, Pavillon L, Edouard Herriot Hospital, Lyon, France; 2 Health Science Licence, Faculté de médecine Lyon Est – Université Lyon 1, Lyon, France; 3 EchOpen Factory, Paris, France

For ecological 1 and economic reasons, the strategies “leave in situ” for hyperplastic polyps and “resect and discard” for diminutive adenomas should be widely adopted to reduce the number of samples unnecessarily sent for pathology examination, resulting in a 319 USD saving per patient 2 and 0.6 kg carbon dioxide equivalent (CO _2e_ ) 3 for each slide of polyp analyzed.

For safe application of those strategies, each physician should demonstrate, with real-life cases, high sensitivity and specificity (>90%) for optical diagnosis of diminutive polyps according to European Society of Gastrointestinal Endoscopy (ESGE) guidelines 4 .

The mobile application “CONECCTapp” (supported by the Société Française d’Endoscopie Digestive) provides an interactive and educational platform designed to support the characterization of colorectal lesions, based on the validated CONECCT classification 5 . The application offers training on previously published lesions through dedicated quizzes ( Fig. 1 **a** ), enabling users to train and consolidate their optical diagnostic skills.

**Fig. 1:**
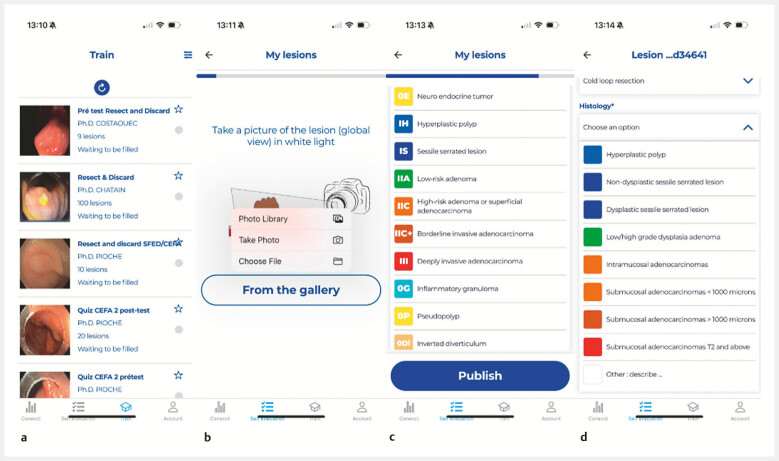
User interface of the CONECCT app. **a** View of the different quizzes for physician training. **b** Different ways to upload a photo. **c** List of CONECCT categories available for physician selection. **d** Histological data entry.

Recently, we developed a new option on the app, with a self-assessment module enabling gastroenterologists to monitor their individual performance in endoscopic characterization (sensitivity and specificity). When they detect lesions, users can upload a photo, input the CONECCT classification on a short form ( Fig. 1 **b, c** , Video 1 ), and specify the strategy chosen between leave in situ, resect and discard, resect and sent for pathology, or sampling for histology in cases of deep invasive cancer. Then, 7 days later, histological results can be added ( Fig. 1 **d** ) into the app to complete each polyp journey.

Example of lesion importation, histological annotation, and diagnostic performance analysis using the CONECCT application.Video 1

Based on this information, the application automatically calculates the sensitivity and specificity of histological prediction ( Fig. 2 **a, b** ). An “action score” is also generated, reflecting the safety of the physician ( Fig. 2 **c** ), with the proportion of polyps adequately left in situ (hyperplastic) or discarded (adenoma) ( Fig. 2 **d** ). After entering at least 120 lesions with sensitivity and specificity over 90%, the gastroenterologist will be considered sufficiently reliable to discontinue routine pathological analysis of certain benign lesions, in line with ESGE guidelines. This module also raises awareness of the environmental impact of endoscopic practices calculating the CO _2e_ saved with a ratio of 0.6 kg CO _2e_ per polyp correctly managed without histology.

**Fig. 2:**
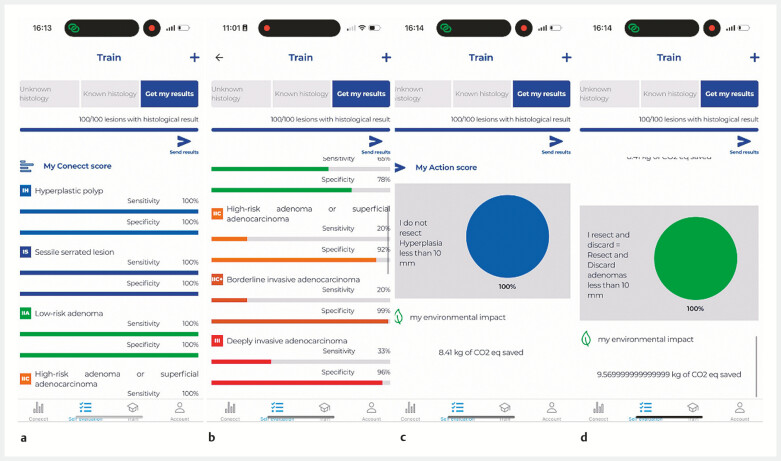
Example screen on the CONECCT app. **a** Simulation of a high diagnostic performance level. **b** Simulation of a lower diagnostic performance level. **c** Action score calculated for the “leave in situ” strategy. **d** Action score calculated for the “resect and discard” strategy.

Adopting a validated, evidence-based, decision-making process could enable more sustainable management of benign lesions, contributing to a measurable reduction in the carbon footprint of colonoscopy. This application brings a new tool to facilitate self-evaluation on real-life practice and promotes a more responsible endoscopy practice – both medically and environmentally.

Endoscopy_UCTN_Code_TTT_1AV

## Notes

Endoscopy E-Videos https://eref.thieme.de/e-videos
*E-Videos* is an open access online section of the journal *Endoscopy* , reporting on interesting cases and new techniques in gastroenterological endoscopy. All papers include a high-quality video and are published with a Creative Commons CC-BY license. Endoscopy E-Videos qualify for HINARI discounts and waivers and eligibility is automatically checked during the submission process. We grant 100% waivers to articles whose corresponding authors are based in Group A countries and 50% waivers to those who are based in Group B countries as classified by Research4Life (see: https://www.research4life.org/access/eligibility/ ). This section has its own submission website at https://mc.manuscriptcentral.com/e-videos .
